# Evaluating PAI-1 as a biomarker for stress in diving: human serum total PAI-1 is unaltered after 2 h dry exposures to 280 kPa hyperbaric air

**DOI:** 10.14814/phy2.12437

**Published:** 2015-06-24

**Authors:** Ingrid Eftedal, Hallvard Aglen Fredriksen, Astrid Hjelde, Andreas Møllerløkken

**Affiliations:** Department of Circulation and Medical Imaging, Faculty of Medicine, Norwegian University of Science and TechnologyTrondheim, Norway

**Keywords:** Circulation, coagulation, diving, inflammation, oxidative stress

## Abstract

Plasminogen activator inhibitor (PAI-1) is induced in the vasculature and secreted into the vascular lumen in response to inflammation and oxidative stress. We have previously reported a fivefold increase in plasma PAI-1 from rats exposed to 708 kPa hyperbaric air. In the current study we assess the potential of human serum total PAI-1 as a biomarker for stress in compressed air diving. Eleven recreational divers, nine males and two females, completed four 2 h hyperbaric air exposures to 280 kPa in a pressure chamber over a period of 2 weeks. The air pressure corresponds to a diving depth of 18 m in water. Serum was collected before the study and again 3 h 30 min after completion of each hyperbaric exposure. All samples were taken in the afternoon to minimize the contribution of circadian variation. The analysis revealed no change in serum total PAI-1 after hyperbaric exposures within the group of divers (*P *=* *0.064), but significant interindividual differences persisted throughout the study (*P *<* *0.0005). A case of decompression sickness after the third round of hyperbaric exposure did not affect PAI-1. In conclusion, compressed air exposure to 280 kPa does not affect serum total PAI-1, and significant interindividual variation in PAI-1 levels may limit its usefulness as a biomarker. This does, however, not give a complete answer regarding PAI-1 in physiologically stressful dives. Further studies with different exposures and timing are needed for that.

## Introduction

In compressed air diving, the diver breathes air at a density equal to that of the surrounding medium. Elevated oxygen pressure during submersion and nitrogen gas released as bubbles during decompression stress the circulatory system and may provoke a variety of symptoms collectively known as decompression sickness (DCS) (Vann et al. 2011). The self-reported incidence of DCS is low (0.155%) (Ranapurwala et al. 2014), but even asymptomatic air dives are associated with proinflammatory responses, augmented oxidative stress (Eftedal et al. 2013; Sureda et al. 2014) and transient endothelial dysfunction (Brubakk et al. 2005). The rareness of DCS speaks of a vital balance between the disruptive propensity of hyperbaric environments and the individual diver's capacity for biological compensation, and factors that drive this balance may be suitable biomarkers for the assessment of physiological stress in diving.

We have previously reported that exposure to 708 kPa compressed air, corresponding to a water depth of 60 m, activates target genes for redox-sensitive transcription factors in the rat aorta (Eftedal et al. 2012). Interestingly, these gene expression changes occurred independently of decompression-induced bubbles, as there were no differences between the responses of animals without detectable vascular bubbles compared to those with high bubble loads in their venous circulation after decompression. Among the upregulated genes was Serpine1, which codes for the serine protease inhibitor PAI-1. The Serpine1 activation was followed by a fivefold increase in PAI-1 protein in blood. PAI-1 is a central regulator of fibrinolysis; in unstressed cells PAI-1 preserves the basal fibrinolytic activity by inhibiting plasminogen activation by tissue-type and urokinase-type plasminogen activator (t-PA and u-PA) (Eitzman et al. 2000). PAI-1 activity is tightly regulated at the transcriptional level, and there is constitutive PAI-1 expression in a variety of cell types, including endothelial cells, smooth muscle cells, macrophages, hepatocytes, adipocytes, and platelets (Booth et al. 1988; Brogren et al. 2004; Dellas and Loskutoff 2005).

Oxidative stress enhances Serpine1 gene expression beyond basal levels in vascular cells (Vulin and Stanley 2004; Liao et al. 2007), and triggers the release of PAI-1 into the vascular lumen (Kawasaki et al. 2000). In addition to its direct prothrombotic function, PAI-1 also stimulates the formation of procoagulant microparticles (Brodsky et al. 2002). It is plausible that high PAI-1 in response to oxidative stress is beneficial from an evolutionary viewpoint: proinflammatory cytokines activate their responses via oxidative stress signaling, and an elevation of PAI-1 may promote coagulation as a protective response, possibly limiting the spread of infections or excessive bleeding in traumatic injury (Esmon et al. 2011). There is, however, a flip side. Elevated PAI-1 over time promotes atherosclerosis (Binder et al. 2002), and is associated with the risk of several diseases, among them thrombotic vascular diseases (Eitzman et al. 2000; Kohler and Grant 2000), and aseptic bone necrosis (Jones et al. 2003).

In this study we examine PAI-1 as a potential biomarker for physiological stress in exposure to hyperbaric air. Based on our previous observations in rats, we hypothesized that total PAI-1 in human serum would be elevated after dry pressure chamber dives. The study protocol was chosen to reflect total and oxygen pressures, exposure times, and decompression stress that may be encountered in recreational diving, so that the results would be relevant to divers at large.

## Materials and Methods

### Research ethics

The study was approved in advance by the Norwegian Regional Committee for Medical and Health Research Ethics (REK approval no 2013/2251), and conducted in compliance with the Declaration of Helsinki ethical principles for human experimentation. Participation was based on written informed consent. The participants were provided with Divers Alert Network (DAN) Europe divers insurance for 1 year from the start of the study. A diving physician was on-call in the vicinity of the study site during the hyperbaric exposures and postexposure observations, and the pressure chamber was set up for hyperbaric oxygen treatment (US Navy table 6) in case any participant developed symptoms of DCS.

### Study participants

Eleven currently active recreational divers, two females and nine males, aged 20 to 47 years (34 ± 6 years, mean ± SD) were included in the study after passing a medical examination based on the criteria for health clearance of inshore occupational divers from the Norwegian Board of Health (Norwegian guidelines for medical examination of occupational divers 2000). In addition to recording of sex, age, weight, and height, the examination included measurements of pulmonary function (spirometry, data not shown), maximal oxygen uptake (VO_2_max treadmill test), and serum total cholesterol, low-density lipoprotein cholesterol (LDL-C) and high-density lipoprotein cholesterol (HDL-C). During the examination one diver reported a previous incidence of possible DCS for which he had received hyperbaric oxygen treatment without consulting a physician 1 year earlier; no other cases of prior diving-related injury were reported among the participants. After inclusion, participation in any specific hyperbaric exposure during the study was contingent the diver not having obstructive symptoms of the upper respiratory tract as this might cause difficulty with middle ear pressure equilibration.

### Hyperbaric exposure

The hyperbaric exposures were performed in a 13,000 L pressure chamber (Dräger, Drägerwerk Lübeck, Germany), localized at Høvringen outside Trondheim and managed by pressure chamber operators from the local fire and rescue service. The participating divers were randomly assigned to four groups, each consisting of 2 or 3 persons. Two groups were scheduled for hyperbaric exposure on each study day; one group before lunch (9.30 to 11.30 am) and one group after lunch (12 to 2 pm). For each participant, singular exposures were set 72 or 96 h apart in order to abolish any transient responses between exposures (Obad et al. 2007). The dive profiles followed the Norwegian diving and treatment tables version 3 (Arntzen et al. 2004) with a bottom pressure of 280 kPa, corresponding to 18 m of sea-water. Compression to 280 kPa was done at 150 kPa·min^−1^. After the divers were kept at a bottom pressure of 280 kPa for 100 min, decompression was performed at a rate of 90 kPa·min^−1^ with two decompression stops: the first at 160 kPa for 5 min, and the second at 130 kPa for 15 min. While in the chamber, the divers had visual and audial contact with the chamber operators at all times.

### Blood sampling and serum protein analyses

In order to limit the effects of circadian variation on biomarker levels, the timing was set so that all blood samples would be drawn in the afternoon between 3 and 5.30 PM, when PAI-1 would be at a relatively stable minimal daily level (Scheer and Shea 2014). Blood for analyses was collected on 5 mL Serum Sep Clot Activator + Gel tubes (Vacuette, Greiner Bio-One International GmbH, Kremsmuenster, Austria) by standard venipuncture at the pre dive-medical examination, and again 3 h 30 min after completion of each hyperbaric exposure. After inversion, the blood tubes were left to clot for 30 min at room temperature before centrifugation at 10,000 g for 10 min. Serum was stored at −80°C until analyzed. Four individual samples were missing, either because the diver was not present for testing (preexposure diver 7, and post2 diver 3), or exclusion from individual dives due to symptoms of common cold (post1 diver 9), and decompression sickness (post4 diver 3).

Total PAI-1, comprising the active and latent forms of PAI-1 in serum, was measured with the human total Serpin E1/PAI-1 Quantikine ELISA Kit from R&D Systems (catalog no DTSE100, Minneapolis, MN). The producer had validated the reagents for serum values of 35.8–137 ng mL^−1^, and by titration we determined a 25-fold dilution of serum samples used in the final analysis. The samples were run in duplicates according to the manufacturer's protocol. OD values were measured at 450 mn using microplate reader (Asys Expert Plus, Biochrom Ltd., Cambridge, UK), and the total PAI-1 concentration was calculated from the standard curves using sample mean OD values with blank correction.

Serum cortisol was measured to assess general stress levels that might affect cardiovascular function (Lazzarino et al. 2013). The analysis was done using the Cobas human cortisol ECLIA kit (catalog no 11875116160) on a Modular E 170 immunochemistry analyzer, both from Roche Diagnostic (Hoffmann-La Roche Ltd, Basel, Switzerland).

### Statistical analysis

A prospective sample size power calculation for PAI-1 analysis was done using IBM SPSS Sample Power (Version 3, IBM Corp., Armonk, NY). Eleven participants provided an estimated power of 80% with an *α* error probability of 0.05 based on plasma PAI-1 data from a general adult population (Margaglione et al. 1998).

All statistical data analyses were performed using IBM SPSS Statistics software (Version 21.0. IBM Corp.). Kolmogorov–Smirnov test and Q–Q normal probability plots verified that the biomarker measurements were normally distributed; thus parametric tests were used in further analyses. A linear mixed model was employed to assess the effect of repeated hyperbaric exposures on the level of total PAI-1 and cortisol, with time of sampling as a fixed categorical factor and diver id as a random factor. One-way analysis of variance (ANOVA) was used to identify statistical differences in serum biomarker levels between the divers. The level of significance was set at *P *≤* *0.05.

## Results

### Individual diver characteristics

Individual anthropometric, blood cholesterol, and fitness data for the participating divers are shown in Table1. According to BMI derived from the participants’ weight and height, six divers were of normal weight, one was underweight, and four were overweight. None were obese. The serum cholesterol values: total cholesterol, HDL-C, and LDL-C, were all within normal range relative to the participants’ sex and age. All participants had VO_2_max values within the age-dependent requirements for occupational diving from the Norwegian Board of Health (Norwegian guidelines for medical examination of occupational divers 2000).

**Table 1:** Individual diver anthropometrics, cholesterol and fitness data.

Diver id	Sex	Anthropometrics			Serum cholesterol [mmol·L^−1^]^1^			Aerobic fitness	
		Age (years)	Weight (kg)	Height (cm)	Total	HDL	LDL	VO_2_max^2^	Peak HR^3^
1	M	20	55.5	185	3.8	1.2	2.3	56.2	213
2	M	36	79.5	180	5.3	1.1	3.5	49.6	188
3	M	39	76.0	172	4.0	0.9	2.4	49.1	182
4	F	47	60.9	157	4.6	2.2	1.0	34.0	183
5	M	27	74.9	186	4.6	1.4	2.5	61.2	235
6	F	42	88.0	174	4.8	1.7	2.7	37.7	184
7	M	32	95.5	189	5.9	1.3	3.8	53.2	185
8	M	32	83.1	185	4.1	1.2	2.0	51.1	198
9	M	44	93.5	183	6.1	1.3	3.9	46.5	196
10	M	30	69.4	175	4.8	1.3	2.9	62.3	188
11	M	22	65.3	180	4.4	2.1	1.9	57.4	194
	Mean	34	76.5	179	4.8	1.4	2.6	50.8	195
	SD	9	13.0	9	0.7	0.4	0.9	8.9	16

1 All cholesterol values are within normal range for the participants’ sex and age.

2 Maximum rate of oxygen consumption measured during treadmill exercise, mL·min^−1^ kg^−1^.

3 Heart rate, min^−1^ at VO_2_max.

### Serum total PAI-1 and cortisol

**Figure 1:**
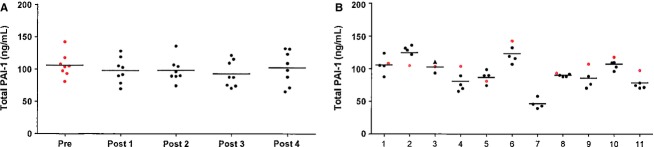
Serum total PAI-1. (A) Serum total PAI-1 according to the time of measurement. Only divers for whom all five samples were available are included (*n* = 8). There are no differences in serum total PAI-1 before and after hyperbaric exposures within the group. (B) All serum total PAI-1 values measured are sorted according to individual diver id (*n* = 11). There is significant interindividual variation in serum total PAI-1 between divers (*P *<* *0.0005). A triangle (▴) marks data from diver 3 for the hyperbaric exposure preceding DCS symptoms. In both panels, red symbols are used for predive values, black for postdive values. Horizontal lines indicate means.

**Figure 2:**
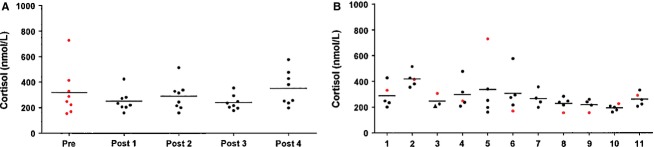
Serum cortisol. (A) Values are sorted according to the time of measurements. Only divers for whom all five samples were available are included (*n* = 8). (B) All serum cortisol measured sorted by individual diver id (*n* = 11). There are no differences in serum cortisol within the group, or between individual divers. In both panels, red symbols are used for predive values, black for postdive values. Horizontal lines indicate means.

Serum total PAI-1 and cortisol measured before the study, and again 3 h 30 min after each of the four hyperbaric exposures, are presented in Figures1 and 2. Figures1A and 2A shows that single or repeated hyperbaric exposures did not affect serum levels of total PAI-1 (*P *=* *0.064) or cortisol (*P *=* *0.286). However, as shown in Figure1B, PAI-1 varied significantly between individual divers (*P *<* *0.0005). Cortisol levels were not different between the divers, Figure2B (*P *=* *0.122). Diver number 3 developed symptoms of DCS approximately 24 h after the third hyperbaric exposure; this had no effect on serum total PAI-1 or cortisol levels 3 h 30 min after this exposure. The participant, not the same diver who reported having experienced DCS 1 year earlier, was excluded from the rest of the study with no further measurements done. He was diagnosed with neurological DCS and received treatment and follow-up according to medical procedures.

## Discussion

No changes in serum total PAI-1 were observed in response to a series of hyperbaric air exposures to 280 kPa in this study. A case of DCS after the third round of hyperbaric exposures indicates that the stress was significant, but the affected diver had unaltered PAI-1 levels also in the measurement that preceded his symptoms.

The incentive for examining PAI-1 as a stress biomarker in diving came from rat studies where hyperbaric air triggered a large increase in plasma PAI-1 independently of whether vascular bubbles were present after decompression (Eftedal et al. 2012). Rodents are convenient models for exposures that lie beyond the limits for safe human diving, and the rat exposures were more provocative than those reported here with regards to partial pressure of oxygen (*pp*O_2_) and decompression stress: the *pp*O_2_ at maximum pressure was 149 kPa in the rat study versus 59 kPa in the present study. A linear decompression 50 kPa·min^−1^ was used for the rats; versus staged decompression following an established diving protocol in the present study. These differences may explain the divergent results. Also, the rats were all of equal genetic and environmental background. The divers in the present study were approved for participation on basis of medical criteria for occupational diving, but as shown in Table1 their individual traits varied considerably. Even if PAI-1 levels positively correlates with long-term risk for a number of cardiovascular diseases, its usefulness as a biomarker for transient physiological stress in healthy subjects may be limited by the normal variability in human PAI-1. Basal PAI-1 levels depends significantly on, for example, sex, age, obesity, cholesterol levels, and physical fitness (Margaglione et al. 1998; Alessi and Juhan-Vague 2006; Cesari et al. 2010; Lira et al. 2010), which may explain the observed interindividual differences in serum total PAI-1. Common genetic variants of the Serpine1 gene are also thought to affect PAI-1 protein levels (Margaglione et al. 1998); these are, however, outside the scope of this study.

In addition to its potential value as a stress biomarker, PAI-1 has a direct role in the pathophysiology of diving. Aseptic bone necrosis is an established complication in frequent and deep diving (Aseptic Bone Necrosis in Commercial Divers 1981; Cimsit et al. 2007), and high PAI-1 has been found to be an independent predictor of bone necrosis in male occupational divers (Miyanishi et al. 2006). If PAI-1 is elevated by diving in itself, as indicated by our earlier rat study, this may be detrimental to the diver's skeletal health in a long-term perspective. Both our previous report on rats and the current study on human divers are, however, conflicting with a previous study in which PAI-1 in human plasma was decreased after 30 min compressed air exposures to 400 and 700 kPa (Radziwon et al. 2007). However, when comparing the Radziwon study and the current, in addition to the differences in material for analysis, for example, serum versus plasma, there are protocol differences that may explain the discrepancy. Beside the differences in hyperbaric exposure, the timing of the PAI-1 measurements was dissimilar. In the current study, the timing of blood sampling was decided on the basis of two factors. First, there is a pronounced circadian variation in PAI-1 levels in human blood (Angleton et al. 1989; Scheer and Shea 2014), with values peaking in the early morning, falling toward mid-day, and remaining relatively stable in the afternoon and evening. We therefore did all blood sampling in the afternoon, whereas the preexposure samples for the Radziwon study appear to have been drawn on the day of exposure. Second, the time it takes from upregulation of the Serpine1 gene in vascular cells to detectable secretion of PAI-1 into the vascular lumen is a factor to consider. In vitro studies indicate that several hours are needed before upregulated gene transcription results in a detectable rise in secreted extracellular PAI-1 (Handt et al. 1996; Jeong et al. 2011). In the current study, 5 h 30 min separated the start of the hyperbaric exposures and the postexposure blood sampling, which probably puts us in the early phases of a potential rise in PAI-1. Even later measurement might have revealed an increase, but for practical reasons this was not done.

### Limitations

Most of the circulating PAI-1 in healthy individuals is contained within platelets (Booth et al. 1988). The aim of this study was to identify stress-induced changes in PAI-1 presumably secreted from the vasculature; serum was chosen to avoid contributions from platelets that might mask smaller changes. There is a risk of platelet lysis with release of PAI-1 during serum preparation. However, the stability of the intraindividual measurements and consistent interindividual differences in total PAI-1 throughout the study leads us to believe that the serum results reflect genuine biology.

The hyperbaric exposures in this study were done in a pressure chamber, and there are some notable differences in the cardiovascular responses to breathing hyperbaric air in a dry environment and diving in water. Among them is the altered distribution of blood from the extremities to the chest cavity during water immersion, and constriction of peripheral blood vessels in response to cold water. The resulting increases of the heart's work-load and blood pressure contribute to the physiological stress in diving, and are not addressed in this study.

## Conclusion

There is no effect of exposure to 280 kPa hyperbaric air on total PAI-1 levels in human serum. Significant interindividual variation in basal PAI-1 levels is likely to limit its usefulness as a biomarker for physiological stress in recreational diving. This does, however, not provide a complete answer regarding the usefulness of PAI-1 measurements in diving. Further studies with different exposures and timing are needed for that.
